# Simple and safe digitoxin dosing in heart failure based on data from the DIGIT-HF trial

**DOI:** 10.1007/s00392-023-02199-z

**Published:** 2023-04-22

**Authors:** Udo Bavendiek, Anika Großhennig, Johannes Schwab, Dominik Berliner, Xiaofei Liu, Lars Maier, Thomas Gaspar, Andreas Rieth, Sebastian Philipp, Rainer Hambrecht, Ralf Westenfeld, Thomas Münzel, Sebastian Winkler, Martin Hülsmann, Dirk Westermann, Marja Zdravkovic, Ralf Lichtinghagen, Heiko von der Leyen, Silke Zimmermann, Christian Veltmann, Michael Böhm, Stefan Störk, Armin Koch, Johann Bauersachs

**Affiliations:** 1 Department of Cardiology and Angiology, Hannover Medical School, Carl-Neuberg-Straße 1, 30625 Hannover, Germany; 2 Institute of Biostatistics, Hannover Medical School, Hannover, Germany; 3 Department of Cardiology, Paracelsus Medical University, Nuremberg, Germany; 4 Department for Internal Medicine II, University Hospital Regensburg, Regensburg, Germany; 5 Department of Internal and Cardiovascular Medicine, Herzzentrum Dresden, University Clinic, Technische Universität Dresden, Dresden, Germany; 6 Department of Cardiology, Kerckhoff-Klinik, Bad Nauheim, Germany; 7 Department of Internal Medicine, Cardiology and Intensive Care Medicine, Elbeklinikum Stade, Stade, Germany; 8 Department of Internal Medicine II, Cardiology, Angiology and Intensive Care Medicine, Klinkum Links Der Weser, Bremen, Germany; 9 Division of Cardiology, Pulmonology and Vascular Medicine, Medical Faculty, University Hospital Duesseldorf, Heinrich-Heine University Duesseldorf, Duesseldorf, Germany; 10 University Medical Center Mainz, Center of Cardiology, Johannes Gutenberg University, Mainz, Germany; 11 Department of Internal Medicine, BG Klinikum Unfallkrankenhaus Berlin, Berlin, Germany; 12 Universitätsklinik Für Innere Medizin II, Abteilung Kardiologie, Medizinische Universität Wien, Vienna, Austria; 13 Department of Cardiology and Angiology, University Heart Center Freiburg—Bad Krozingen, Faculty of Medicine, University of Freiburg, Freiburg, Germany; 14 University Hospital Medical Center Bezanujska Kosa, Belgrade, Serbia; 15 Institute for Clinical Chemistry, Hannover Medical School, Hannover, Germany; 16 Orgenesis, Inc, Germantown, USA; 17 Center for Clinical Trials, Hannover Medical School, Hannover, Germany; 18 Center for Electrophysiology Bremen, Bremen, Germany; 19 Klinik Für Innere Medizin III, Universitätsklinikum Des Saarlandes, Saarland University, Homburg a. d. Saar, Germany; 20 Department Clincical Reserch & Epidemiology, Comprehensive Heart Failure Center, University Hospital Würzburg, Würzburg, Germany

**Keywords:** Cardiac glycosides, Digitoxin, Heart failure, Clinical trial, Dose titration

## Abstract

**Background:**

The present study aimed to develop a simple dosing score when starting the cardiac glycoside digitoxin in heart failure with reduced ejection fraction (HFrEF) employing first data from the randomized, double-blinded DIGIT-HF trial.

**Methods and results:**

In DIGIT-HF, digitoxin was started with a dose of 0.07 mg once daily (o.d.) in all patients. For score derivation, 317 patients were analyzed who had been randomized to digitoxin. In these patients, after scheduled determination of serum levels at study week 6, the digitoxin dose had remained unchanged or had been reduced to 0.05 mg o.d. (97% of patients) to achieve serum concentrations within a predefined range (10.5–23.6 nmol/l). In logistic regression analyses, sex, age, body mass index (BMI), and estimated glomerular filtration rate (eGFR) were associated with need for dose reduction and, therefore, selected for further developing the dosing score. Optimal cut-points were derived from ROC curve analyses. Finally, female sex, age ≥ 75 years, eGFR < 50 ml/min/1.73 m^2^, and BMI < 27 kg/m^2^ each were assigned one point for the digitoxin dosing score. A score of ≥ 1 indicated the need for dose reduction with sensitivity/specificity of 81.6%/49.7%, respectively. Accuracy was confirmed in a validation data set including 64 patients randomized to digitoxin yielding sensitivity/specificity of 87.5%/37.5%, respectively.

**Conclusion:**

In patients with HFrEF, treatment with digitoxin should be started at 0.05 mg o.d. in subjects with either female sex, eGFR < 50 ml/min/1.73m^2^, BMI < 27 kg/m^2^, or age ≥ 75 years. In any other patient, digitoxin may be safely started at 0.07 mg o.d.

**Graphical Abstract:**

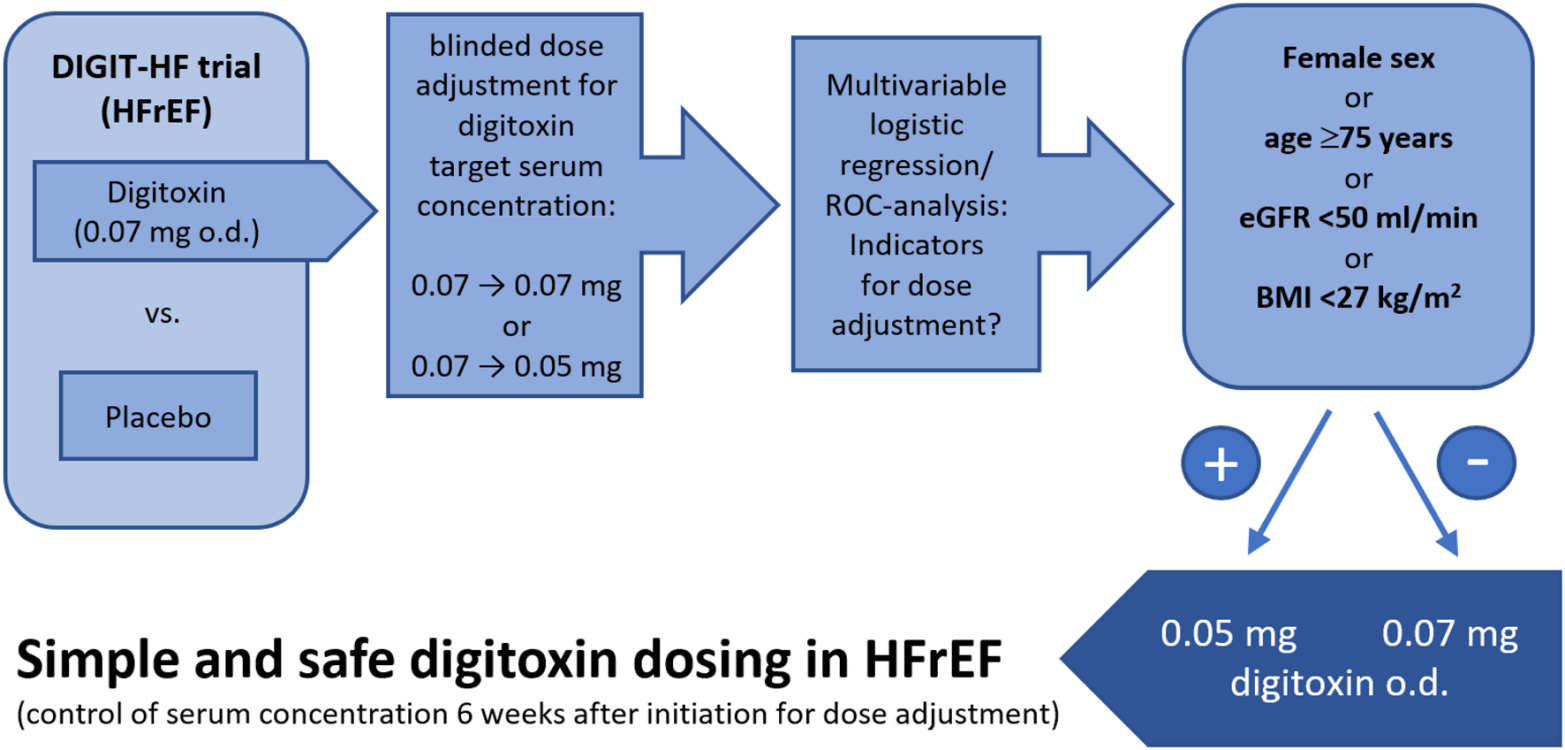

## Introduction

Cardiac glycosides represent a valuable therapeutic option for patients with heart failure and a reduced ejection fraction (HFrEF), who remain symptomatic despite optimized guideline-directed pharmacotherapy and device therapy [1]*.* Further, current guidelines recommend cardiac glycosides for rate control in atrial fibrillation (AF) in patients with and without heart failure [2, 3]. However, prescription rates of cardiac glycosides have been declining due to complementary pharmacologic therapies with beta-blocker, angiotensin-converting enzyme inhibitors (ACEi)/angiotensin-receptor-blocker (ARB), mineralocorticoid receptor antagonists (MRA), angiotensin receptor neprilysin inhibitors (ARNI), and sodium-glucose-cotransporter-2 inhibitors (SGLT2i) improving mortality and morbidity in patients with HFrEF [4]. In addition, the relative narrow therapeutic range of cardiac glycoside blood concentrations caused concerns of overdosing and toxicity. Recommendations for simple and safe dosing of cardiac glycosides in clinical practice could enable safer use of cardiac glycosides in patients with HFrEF and/or AF.

Both digoxin and digitoxin are approved cardiac glycosides and most frequently used for the treatment of patients with HFrEF and/or AF. Information about the relationship of cardiac glycoside dose and blood concentration in HFrEF and/or AF based on clinical trial data is only available for digoxin [3, 5], but not for digitoxin. However, digitoxin represents the pharmacokinetically more stable cardiac glycoside compared with digoxin, especially in patients with impaired renal function [6, 7]. Therefore, digitoxin might even exceed potential beneficial effects observed with digoxin in patients with HFrEF and/or AF [3, 5].

The DIGIT-HF trial is a randomized, double-blind placebo-controlled trial investigating the impact of digitoxin on mortality and hospitalizations for worsening heart failure in patients with advanced HFrEF [8]. Importantly, patients with AF or advanced impairment of renal function were not excluded. Due to the pharmacologic properties of digitoxin, a simple standardized dose titration protocol was applied that aimed to achieve serum concentrations within the lower therapeutic range that is currently recommended for cardiac glycosides based on data from the DIG trial [5]. The current analysis from the DIGIT-HF trial population sought to identify the factors influencing the relationship of digitoxin dose decisions on serum concentrations. Based on this analysis, a score was developed, which can easily be used in clinical practice to select initial digitoxin dosing to avoid overdosing.

## Methods

### Cohort description

The DIGIT-HF trial is a multicenter, randomized, double-blind, placebo-controlled investigator initiated clinical trial designed to demonstrate the efficacy of digitoxin in patients with HFrEF. Details on the design of the DIGIT-HF trial have been provided elsewhere [8]. Digitoxin treatment was titrated by a standardized dosing protocol to achieve serum concentrations within a predefined low therapeutic range of 10.5–23.6 nmol/l. Patients randomized to digitoxin or matching placebo started with a dose of 0.07 mg once daily (o.d.). For dose titration, digitoxin serum concentrations were determined in a blinded fashion for all patients randomized to digitoxin or placebo 6 weeks after randomization (visit 1) in a core laboratory, and dose adjustments are centrally initiated to avoid unblinding. Initiation of digitoxin treatment with the daily maintenance dose and without loading will produce steady state levels not until 3–4 weeks [9]. Therefore, to ensure stable digitoxin serum concentrations for standardized dose titration, 6 weeks after start of treatment was chosen as the point of time for determination of digitoxin serum concentrations. In the digitoxin group, dose adjustment employs a predefined algorithm. If digitoxin serum levels are outside the target range of 10.5–23.6 nmol/l, doses are reduced or increased to 0.05 or 0.1 mg digitoxin o.d., accordingly. Otherwise, the starting dose of 0.07 mg digitoxin o.d. is maintained. In the placebo group, dose adjustment is randomly assigned. Within the standardized dosing protocol, patients did not receive additional loading doses per-protocol during the trial.

**Fig. 1:**
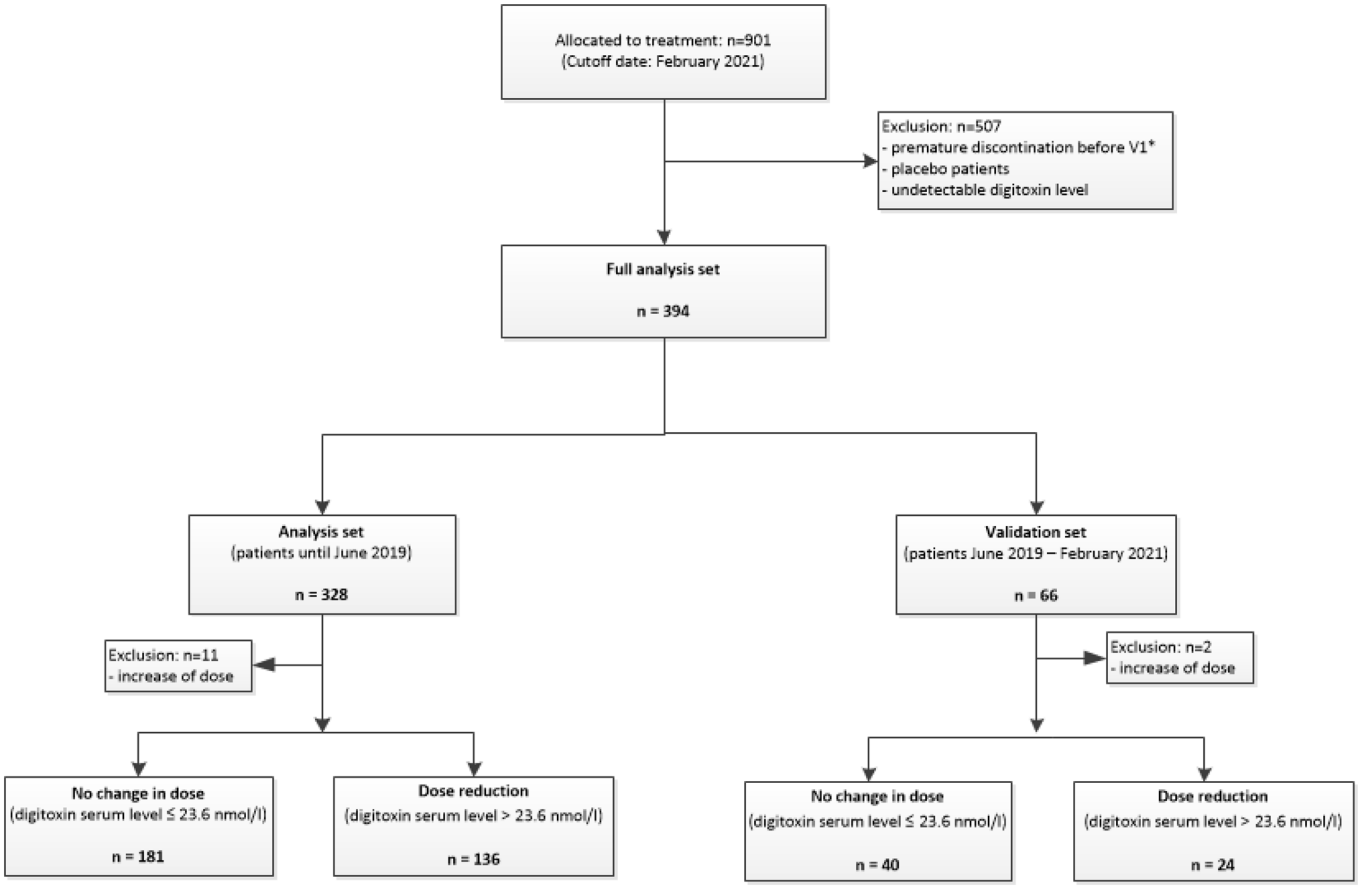
Overview of selection of analysis population. * including deaths, adverse or serious adverse events leading to exclusion for further study participation, exclusion due to decision of the principal investigator, withdrawal of informed consent, lost to follow-ups, other or unknown (not documented) reasons

The current analyses are based on 901 patients, who had been randomized to digitoxin or placebo in the DIGIT-HF trial prior to March 2021. For the analysis in this manuscript, only patients having an available digitoxin level and a dose adaptation at week 6 could be analyzed. Therefore, patients which have been randomized to placebo or had no available digitoxin level and dose adaptation at week 6 for different reasons (death, adverse or serious adverse events leading to exclusion for further study participation, exclusion due to decision of the principal investigator, withdrawal of informed consent, lost to follow-up, other or unknown (not documented) reasons, undetectable digitoxin level due to potential compliance problems) were excluded from this analysis. Finally, 394 patients randomized to digitoxin with available digitoxin levels and dose adaptations at week 6 could be used for the present analysis. A total of 221 patients (56%) maintained a starting dose of 0.07 mg o.d. In 160 patients (41%), the digitoxin dose was reduced from 0.07 mg to 0.05 mg o.d. In 13 (3%) patients, the digitoxin dose was increased from 0.07 mg to 0.1 mg o.d. Patients up-titrated from 0.07 mg to 0.1 mg digitoxin o.d. were excluded, yielding a total of 381 patients suitable for this analysis (i.e., allocation to the digitoxin arm and continuation or reduction of digitoxin starting dose based on digitoxin serum concentrations measured at visit 1). These patients were split into a derivation set (*n*_analysis_ = 317 patients recruited until June 2019) and a validation set (*n*_validation_ = 64 patients recruited between July 2019 and February 2021). Patients were grouped by the necessity of a dose reduction. Specifically, patients continuing the initial dose of 0.07 mg o.d., because of a digitoxin serum level ≤ 23.6 nmol/l (*n*_analysis_ = 181/*n*_validation_ = 40), were compared with patients receiving a dose reduction to 0.05 mg o.d. because of a digitoxin serum level > 23.6 nmol/l or even the recommendation of discontinuation due to a digitoxin serum level ≥ 33 nmol/l (*n*_analysis_ = 136/*n*_validation_ = 24). The specific selection of the analysis and the validation data set is depicted in Fig. 1.

### Determination of digitoxin serum concentrations

Serum samples drawn at study visit 1 (6 weeks after randomization) were sent to a central core laboratory (Dept. of Clinical Chemistry, Hannover Medical School) for determination of digitoxin serum concentrations employing a standardized assay (Elecsys Digitoxin cobas®, Roche Diagnostics GmbH).

### Statistical methods

#### Building the digitoxin dosing score

First, we identified variables associated with a higher chance of a digitoxin dose reduction. Therefore, we performed univariable two-sided *χ*^2^-test for categorical parameters and two-sided *t* tests for continuous variables selected from a larger panel of baseline variables of the DIGIT-HF study, based on clinical reasoning and findings reported in the literature. In the second step, we performed an univariable logistic regression analysis including all parameters with a two-sided *p* value smaller than 5%. For the identification of the most relevant factors, we performed a multivariable logistic regression analysis applying a backward selection procedure with *p* value criteria for staying in the model of 5%. To identify optimal thresholds for continuous parameters, we performed receiver operating characteristic (ROC) analyses in the next step. Optimal cut-points based on the Youden index were estimated and rounded with respect to clinical meaningful thresholds. Finally, all relevant parameters were equally weighted and the score was built as a sum of the single components. To indentify the score with the highest propability of identifying the patients that correctly received a dose reduction, different combinations of score variables and sum scores were again analyzed using univariable and multivariable logistic regression analyses.

#### Accuracy of the score

To assess the decisions for the necessity of a dose reduction (yes or no) derived from the dichotomized score, we calculated the sensitivity and specificity in the derivation and validation sets. Sensitivity was defined as the probability that a patient needing dose reduction had been correctly identified, i.e., score ≥ 1. Specificity was defined as the probability that a patient needing no dose reduction had been correctly identified, i.e., score = 0.

All analyses were performed with SAS 9.4. Strict blinding regarding individual information on treatment allocation and outcome was ascertained as only one unblinded statistician performing the statistical analyses had access to the data set and all results were displayed to authors only on an aggregated basis.

### Ethics

The DIGIT-HF trial is conducted in compliance with the German Drug Law (AMG), the German Good Clinical Practice (GCP) ordinance, ICH GCP guidelines, and other applicable ethical and regulatory requirements. The DIGIT-HF trial is registered at EudraCT (2013-005326-38).

## Results

### Variables associated with digitoxin dose reduction

First, we descriptively compared baseline variables of the derivation set (*n* = 317) between the two groups of patients with and without necessity of digitoxin dose reduction identified 6 weeks after randomization. Dose reductions occurred more often in female or older patients, patients with lower body mass index (BMI), lower estimated glomerular filtration rate (eGFR based on the CKD-EPI formula) [10], higher serum urea or creatinine concentrations, lower blood leukocyte count, and less frequent in patients with a history of hypertension, diabetes or treatment with an ARNI (Table 1). In the second step, all these factors were analyzed with univariable and multivariabled logistic regression models using backward selection. There, sex, BMI, and eGFR remained the predominant factors predicting the necessity of digitoxin dose reduction (Table 2) and were, thus, used for compilating the dosing score. Although age missed the significance level in multivariable regression analysis, it was considered for score compilation because of the well-acknowledged lower muscle mass and, therefore, smaller volume of digitoxin distribution observed in the elderly [6].

**Table 1:** Baseline parameters of the analysis set (n = 317)

	Reference group/unit	*n* missing	No dose reduction (*n* = 181)	Dose reduction (*n* = 136)	*p* value *χ*^2^-test/*t* test
**Baseline**					
Sex	Female	0	20 (11.1%)	41 (30.2%)	< .001
Age	Year	0	64.5 ± 10.3	68.1 ± 11.1	0.003
Body mass index	kg/m^2^	1	31.2 ± 6.0	27.6 ± 5.0	< .001
Smoking	Yes	13	60 (35.5%)	51 (37.8%)	0.590
	Former smoker		34 (20.1%)	21 (15.6%)	
	No		75 (44.4%)	63 (46.7%)	
**Cardiac function**					
Ejection fraction	**%**		28.9 ± 6.4	27.8 ± 7.1	0.130
NYHA functional class	II	0	120 (66.3%)	103 (73.5%)	0.167
	III or IV		61 (33.7%)	36 (26.5%)	
Sinus rhythm	Yes	0	147 (81.2%)	104 (76.5%)	0.303
Atrial fibrillation	Yes	1	26 (14.4%)	21 (15.4%)	0.805
AV-Block	Yes	5	117 (66.1%)	95 (70.4%)	0.524
	No		32 (18.1%)	18 (13.3%)	
	Irrelevant		28 (15.8%)	22 (16.3%)	
**History of other disorders**					
Cardiomyopathy	Yes	2	92 (51.1%)	70 (51.9%)	0.896
Diabetes	Yes	0	69 (38.1%)	37 (27.2%)	0.041
Hypertension	Yes	0	150 (82.9%)	98 (72.1%)	0.021
Hyperlipidemia	Yes	0	109 (60.2%)	85 (62.5%)	0.680
**Anamestic comorbidities**					
Hypothyreosis	Yes	1	16 (8.8%)	20 (14.8%)	0.098
PAD	Yes	0	14 (7.7%)	19 (14.0%)	0.072
Depression	Yes	0	15 (8.3%)	10 (7.4%)	0.760
Cerebrovascular disease	Yes	0	16 (8.8%)	15 (11.0%)	0.516
COPD	Yes	0	22 (12.2%)	20 (14.7%)	0.507
Cancer	Yes	1	11 (6.1%)	7 (5.2%)	0.714
**HF medication**					
ACE inhibitor	Yes	0	84 (46.4%)	59 (43.4%)	0.592
MRA	Yes	0	134 (74.0%)	96 (70.6%)	0.496
ARNI	Yes	0	51 (28.2%)	24 (17.7%)	0.029
AT1 receptor blocker	Yes	0	45 (24.9%)	46 (33.8%)	0.081
Beta-blocker	Yes	0	174 (96.1%)	134 (98.5%)	0.3094*
Ivabradine	Yes	0	13 (7.2%)	16 (11.8%)	0.161
**Laboratory parameters**					
ALAT	U/l	5	27.8 ± 15.7	26.1 ± 20.0	0.387
ASAT	U/l	12	28.9 ± 12.2	27.2 ± 15.9	0.298
Gamma GT	U/l	8	66.7 ± 80.0	74.2 ± 83.9	0.423
Hemoglobin	g/dl	0	13.8 ± 1.9	13.2 ± 1.8	0.009
Urea	mmol/l	14	8.3 ± 4.7	10.2 ± 5.7	0.002
Potassium	mmol/l	2	4.4 ± 0.5	4.5 ± 0.5	0.083
Sodium	mmol/l	2	140.0 ± 2.9	139.6 ± 3.5	0.226
Creatinine	µmol/l	2	109.2 ± 34.5	128.4 ± 73.3	0.002
eGFR (CKD-EPI)	ml/min/1.73m^2^	2	69.5 ± 21.7	57.4 ± 22.4	< .001
Leukocytes	10^3^/µl	0	8.2 ± 2.1	7.6 ± 2.4	0.024

Compared are patients with and without necessity of a digitoxin dose reduction based on digitoxin serum concentrations determined 6 weeks after randomization and start of study medication. For categorical parameters for the respective reference groups, absolute and relative frequencies and two-sided *p* values of the *χ*^2^-test (* or of the Fisher’s exact test) are presented. For continuous parameters, the unit, means, standard deviations, and two-sided *p* values of the independent *t* test are displayed. *p* values smaller than 0.05 are shaded in gray

*ACE* angiotensin-converting enzyme, *AT1* angiotensin 1, *ARNI* angiotensin receptor neprilysin inhibitor, *ALAT* alanine aminotransferase, *ASAT* aspartate amonitransferase, *COPD* chronic obstructive pulmonary disease, *HF* heart failure, *eGFR* estimated glomerular filtration rate (CKD-EPI), *GT* glutamyltransferase, *MRA* mineralocorticoid receptor antagonist, *PAD* peripheral artery disease

**Table 2:** Results of univariable and multivariable logistic regression analyses

Parameter	Unit	Univariable analysis				Multivariable analysis			Final multivariable model after backward selection			
		OR	95% CI	*p* value	OR		95% CI	*p* value	OR	95% CI		*p* value
Sex (reference male)		3.47	1.92–6.28	< .001	3.22		1.51–7.37	0.003	3.41	1.72–6.73		0.001
Age	Year	1.03	1.01–1.06	0.004	1.00		0.97–1.03	0.954				
Body mass index	kg/m^2^	0.89	0.85–0.93	< .001	0.89		0.85–0.94	< .001	0.88	0.83–0.92		< .001
History of diabetes	Yes	0.61	0.38–0.98	0.042	0.90		0.50–1.63	0.728				
History of hypertension	Yes	0.53	0.31–0.91	0.022	0.53		0.26–1.07	0.074				
ARNI	Yes	0.55	0.32–0.94	0.030	0.62		0.33–1.18	0.147				
Hemoglobin	g/dl	0.85	0.75–0.96	0.011	0.96		0.83–1.11	0.556				
Urea	mmol/l	1.07	1.02–1.12	0.003	0.99		0.92–1.07	0.834				
Creatinine	µmol/l	1.01	1.00–1.02	0.003	1.00		0.99–1.02	0.659				
eGFR (CKD-EPI)	ml/min/1.73 m^2^	0.98	0.97–0.99	< .001	0.98		0.95–1.01	0.170	0.98	0.97–0.99		0.001
Leukocytes	10^3^/µl	0.89	0.80–0.99	0.026	0.90		0.79–1.03	0.118				

All baseline parameters which showed a *p* value smaller than 0.05 in the comparison of the baseline are used in the logistic regression analyses. Displayed are odds ratios (OR) with 95% confidence intervals (CI) and *p* values from logistic regression models

*ARNI* angiotensin receptor neprilysin inhibitor, *CI* confidence interval, *eGFR* estimated glomerular filtration rate (CKD-EPI), *OR* odds ratio

### Estimation of optimal cut-points and multivariate analysis of components

**Fig. 2:**
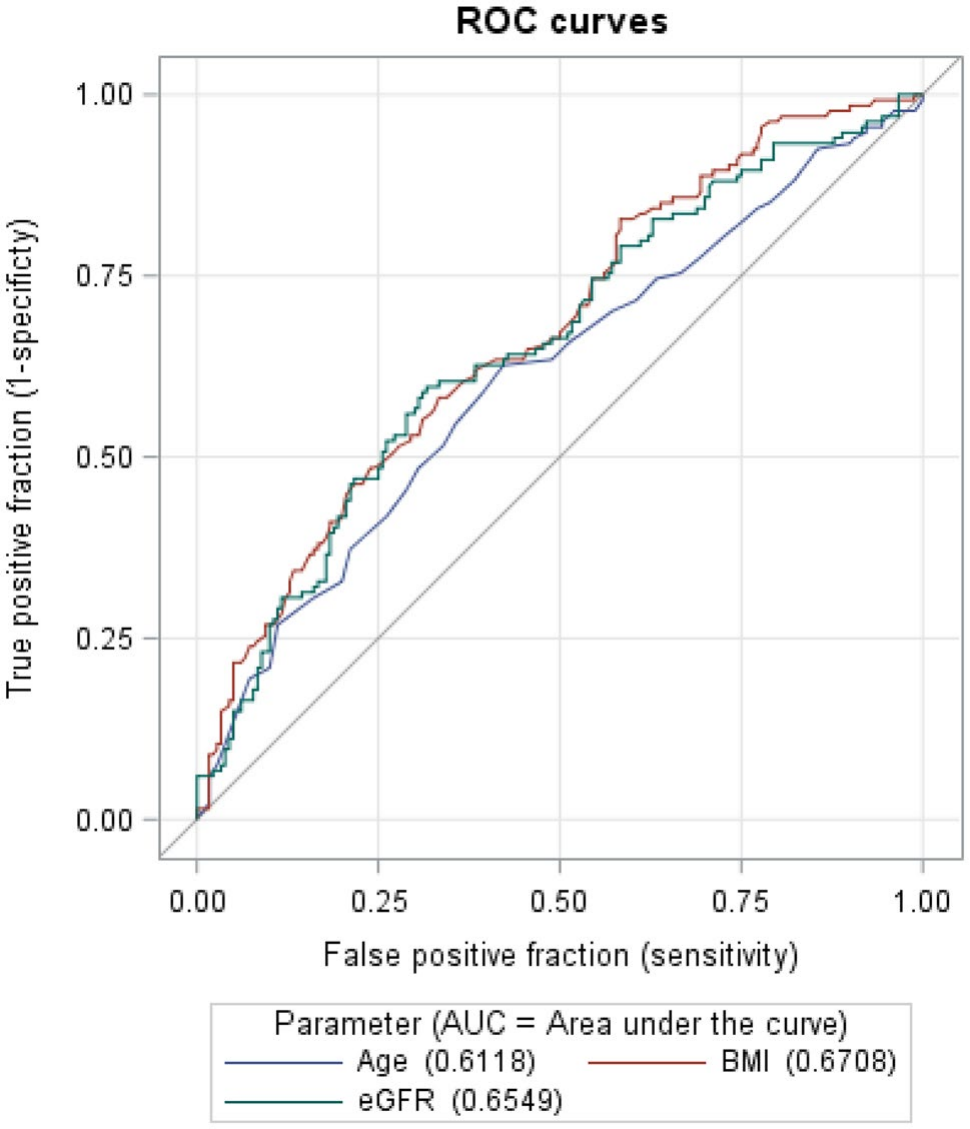
Diagnostic accuracy of dose reduction estimated by ROC curves of age, BMI, and eGFR. *BMI* body mass index, *eGFR* estimated glomerular filtration rate (CKD-EPI), *ROC* receiver operating characteristic

For the continuous parameters, ROC curves were estimated (Fig. 2) and optimal cut-points were derived: age 76 years, BMI 26.9 kg/m^2^, eGFR 51 ml/min/1.73m^2^. As our intention was to develop a simple score, we decided to round up or down to full integers. Since age was kept in the model for clinical reasons, we used the threshold of age ≥ 75 years for the score. The other three components, i.e., female sex, BMI, and eGFR, retained meaningful associations in the multivariable model (Table 3). This yielded a higher probability for the necessity of dose reduction in women (OR 3.20, 95% CI 1.70–6.00), patients with eGFR < 50 ml/min/1.73 m^2^ (OR 2.14, 95% CI 1.21–3.78), or a BMI < 27 kg/m^2^ (OR 3.14, 95% CI 1.90–5.23). In the final model, the effect for age ≥ 75 years was modest and not statistically significant in the multivariable model: OR 1.22, 95% CI: 0.67–2.22 (Table 3).

**Table 3:** Results of the univariable and multivariable logistic regression analysis for digitoxin dosing score and its components

		No dose reduction (*n* = 181)	Dose reduction (*n* = 136)	Univariable regression			Multivariable regression			
				OR	95% CI	*p* value	OR	95% CI		*p* value
Femal sex		20 (11.1%)	41 (30.2%)	3.47	1.92–6.28	< .001	3.20	1.70–6.00		0.001
eGFR (CKD-EPI) < 50 ml/min/1.73 m^2^		38 (21.0%)	56 (41.2%)	2.63	1.61–4.32	0.001	2.14	1.21–3.78		0.009
Age ≥ 75 years		36 (19.9%)	44 (32.4%)	1.93	1.15–3.22	0.012	1.22	0.67–2.22		0.511
BMI < 27 kg/m^2^		44 (24.3%)	67 (49.3%)	3.02	1.88–4.88	< .001	3.15	1.90–5.23		< 0.001
Digitoxin dosing score 0 1 2 3 4		90 (49.7%) 53 (29.3%) 29 (16.0%) 9 (5.0%) 0 (0.0%)	25 (18.4%) 46 (33.8%) 38 (27.9%) 22 (16.2%) 5 (3.7%)	*Referent* 3.13 4.72 8.80 n.c	n.c 1.73–5.66 2.45–9.09 3.60–21.50 n.c	n.c 0.001 < 0.001 < 0.001 n.c				
digitoxin dosing score ≥ 1		91 (50.3%)	111 (81.6%)	4.39	2.60–7.41	< 0.001				

Displayed are absolute and relative frequencies, as well as odds ratios (OR), respective 95% confidence intervals (CI) and *p* values from the logistic regression models

*BMI* body mass index, *eGFR* estimated glomerular filtration rate (CKD-EPI), *n.c.* not computed

### Digitoxin dosing score: definition and performance

**Fig. 3:**
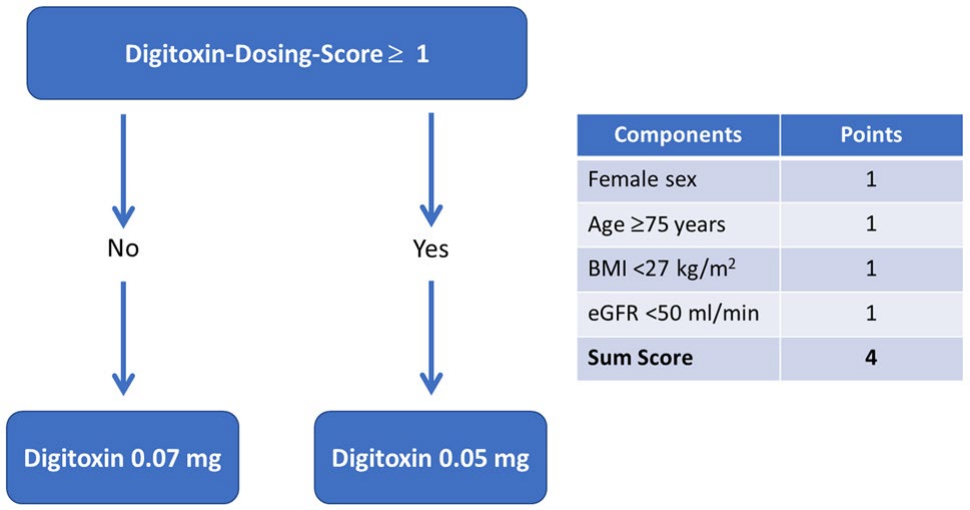
Scheme of digitoxin dosing score and digitoxin dosing at initiation of therapy. *BMI* body mass index, *eGFR* estimated glomerular filtration rate (CKD-EPI)

The calculation scheme of the digitoxin dosing score is depicted in Fig. 3. Female sex, age ≥ 75 years, eGFR < 50 ml/min/1.73 m^2^, and BMI < 27 kg/m^2^ were assigned one point each, rendering a sum score of four points. A low dose of 0.05 mg of digitoxin is recommended if the digitoxin dosing score is ≥ 1. For a digitoxin dosing score of ≥ 1, sensitivity was 81.6% and specificity was 49.7% (Table 4).

**Table 4:** Sensitivity and specificity results of the analysis and validation set

		Dose reduction			Sensitivity	Specificity
		Yes	No	Total		
Analysis set	Digitoxin dosing score ≥ 1	111	91	202	111/136 81.6%	90/181 49.7%
	Digitoxin dosing score = 0	25	90	115		
	Total	136	181	317		
Validation set	Digitoxin dosing score ≥ 1	21	25	46	21/24 87.5%	15/40 37.5%
	Digitoxin dosing score = 0	3	15	18		
	Total	24	40	64		

Displayed are absolute and relative frequencies

#### (Internal) validation

The digitoxin dosing score was applied to 64 patients that had been randomized between July 2019 and February 2021. Table 4 presents the distribution of components and the digitoxin dosing score in the validation set. Here, sensitivity and specificity were 87.5% and 37.5%, respectively (Table 4).

## Discussion

Although the cardiac glycoside digitoxin has been in clinical use for decades, little data are available enabling simple and safe dose titration of digitoxin, especially in patients with HFrEF. DIGIT-HF is the only sizeable randomized clinical trial investigating the effect of digitoxin within a prespecified range of serum concentrations on clinical outcomes in HFrEF [8]. We analyzed dose titration data of a large sample randomized to digitoxin within DIGIT-HF, and identified dominant factors associated with the necessity of digitoxin dose reduction that allowed to derive and validate a digitoxin dosing score. These factors, age, sex, eGFR, and BMI, are readily available in clinical practice. Application of the score allowed to achieve the predefined desirable low therapeutic range of 10.5–23.6 nmol/l, a range equivalent to digoxin concentrations of 0.5–0.9 ng/ml that are currently recommended based on data from the DIG trial. Hence, application of the score is expected to enable safe initiation of digitoxin treatment in clinical routine avoiding overdosing.

The only larger data source available up to now demonstrating a relationship between serum concentrations of cardiac glycoside and outcomes in patients with heart failure is the DIG trial. DIG investigated the effect of digoxin vs. placebo on outcomes in patients in sinus rhythm and LVEF < 45% [5]. Digoxin serum concentrations < 1.0 ng/ml were associated with reduced risks of mortality and heart failure hospitalizations, but concentrations > 1.2 ng/ml were associated with an increased mortality, mainly driven by the increased risk observed in women, but not in men [11–13]. The recently published RATE-AF trial prospectively investigated effects of digoxin on patient-reported quality of life compared to bisoprolol in patients with permanent AF and heart failure symptoms. With a mean digoxin dose of 0.16 mg, mean digoxin serum concentrations were 0.78 ng/ml. Digoxin did not improve the primary quality of life endpoint (SF-36), but significantly improved the secondary outcomes European Heart Rhythm Association (EHRA)-class, New York Heart Association (NYHA) functional class and N-terminal pro-brain-natriuretic-peptide (NT-proBNP) levels, and was associated with fewer adverse events compared to bisoprolol treatment [3].

Adverse effects and toxicity of cardiac glycosides are very rare at serum concentrations in the lower therapeutic range (for digoxin: 0.5–0.9 ng/ml or 0.65–1.15 mmol/L; for digitoxin 8–18 ng/ml or 10.5–23.6 mmol/l), but have been described at serum concentrations > 2.0 ng/ml of digoxin and > 30 ng/ml of digitoxin [14]. Based on the DIG and RATE-AF data, treatment with digoxin within serum concentrations below 1.0 ng/ml seems to be safe in patients with heart failure and/or AF [3, 5]. In the DIG trial, digoxin doses for each patient were chosen with a nomogram taking into account age, sex, body weight, and renal function [15, 16]. However, no data are available in patients with HFrEF demonstrating the relationship between digitoxin dose and blood concentrations.

Because of their different pharmacokinetic properties, digitoxin represents the pharmacologically more stable cardiac glycoside compared to digoxin, especially in patients with impaired renal function [6, 7]. Lipophilic digitoxin shows almost complete enteral absorption with a high bioavailability after oral administration (95–100%) and a high plasma protein binding (90–97%). Skeletal muscle, myocardium, kidney, and liver represent the main compartments of distribution. In the elderly, the distribution volume is considerably lower due to reduced skeletal muscle mass (up to 40%), which represents the major tissue compartment. In contrast to digoxin, which is primarily eliminated renally by passive glomerular filtration and tubular secretion, impaired renal function does not influence elimination and half-life of digitoxin, because the reduced renal clearance is entirely compensated by extrarenal (entero-hepatic) clearance keeping the total clearance constant. Only in patients with advanced liver and renal dysfunction, total clearance of digitoxin is impaired with relevant elevation of digitoxin serum concentrations. Overall, the pharmacokinetic properties of digitoxin ensure stable blood concentrations after reaching a steady state at a given dose even in patients with impaired renal function. In contrast, digoxin blood concentrations are less stable, especially at renal dysfunction, with the need of regular controls of blood concentrations.

In the current analysis, we identified eGFR, BMI, and in particular female sex as factors associated with the necessity of a digitoxin dose reduction in the derivation cohort chosen from the DIGIT-HF population. The strong association with female sex in multivariable regression models might reflect the lower skeletal muscle mass of women compared to men, associated with a reduced compartment and overall digitoxin distribution volume resulting in higher digitoxin serum concentrations in women compared to men for a defined digitoxin dose [6]. Cytochrome P450 IIIA isoenzymes are the main metabolizing enzymes of digitoxin mainly expressed in the liver [17]. As expression of cytochrome P450 IIIA isoenzymes is reduced in women compared to men [17]*,* this may result in a lower digitoxin metabolization and potentially higher serum concentrations in women [18]*.*

The next most significant factor associated with the necessity of a digitoxin dose reduction was BMI. BMI, in general, positively correlates with total skeletal muscle mass. Therefore, the observed negative association between BMI and necessity for digitoxin dose reduction reflects, similar as for female sex, the relationship between the compartment and overall volume of digitoxin distribution, which is higher in patients with a higher BMI resulting in the need for higher digitoxin doses to achieve therapeutic serum concentrations.

Unexpectedly, we also identified a significant negative association between eGFR and necessity of digitoxin dose reduction. The causal relationship is not clear, because digitoxin elimination is entirely compensated by extrarenal (entero-hepatic) clearance even in patients with impaired renal function and digitoxin serum concentrations are constant even if renal function is markedly impaired [6]. Although skeletal muscle mass, the main compartment of digitoxin distribution, is reduced by up to 40% in old age [19], the effect for older patients (age ≥ 75 years) was modest and not statistically significant in the multivariable model, most likely due to collinearity between age and eGFR.

Specifically, the correlation between age and eGFR is -0.55 in our data set, which is substantial. Guided by our aim to maximize the probability of detecting all patients who need a dose reduction, we added age to the score and detected in the analysis and the validation set each three more patients for a dose reduction, which increased score sensitivity (data not shown). Therefore, and taking into account knowledge for the need of digitoxin dose reduction because of reduced muscle mass and, therefore, smaller volume of digitoxin distribution in the elderly [6], age still was considered for dosing score development.

The intention of this analysis was to develop a score that faciliatates the dosing decision when treatment with digitoxin needs to be started in HFrEF patients. The components of the score are readily available and with high validity in clinical routine. Female sex, high age, low BMI, and impaired renal function fulfilled these criteria. From the clinical perspective and patient safety, a dosing score should, in particular, prevent overdosing of digitoxin, because digitoxin serum concentrations exceeding the therapeutic range might cause grave side effects or even toxicity. Also, the long half-life of digitoxin leads to prolonged elimination times rendering overdose a significant clinical problem of digitoxin. Therefore, a respective dosing score should have high sensitivity to detect the need of a dose,reduction from 0.07.mg to 0.05 mg o.d. in the present population, which would encourage to start with the low dose of 0.05 mg digitoxin o.d. in clinical practice. Specificity of the score on the other hand is negligible, because we would recommend measurement of digitoxin serum concentration after 6 weeks to check whether a dose of 0.05 mg digitoxin o.d. was sufficient to reach serum concentrations within the therapeutic range. Based on the digitoxin dosing score, all women, and all patients ≥ 75 years or with a BMI < 27 kg/m^2^ or a eGFR < 50 ml/min/1.73m^2^ should start with 0.05 mg digitoxin o.d.. This approach appears easy to implement and may be expected to improve clinical care. Importantly, the digitoxin dosing score was developed based on randomized, placebo-controlled, double-blinded clinical trial data. However, if this score that we propose here will prove useful in clinicial practise will have to be determined, ideally prospectively in a patient population within standard clinical care.

Our prospective trial data support a retrospective analysis of adverse drug reactions in Germany, indicating adverse drug reactions because of overdosing with digitoxin particular in patients < 70 kg, > 80 years, and women [20]. Despite that, another retrospective analysis reported a lower risk for toxicity in the elderly for digitoxin than for digoxin [21], which may presumably be due to the advantageous pharmacokinetic properties of digitoxin compared to digoxin as described above. Our analysis identified eGFR as an important parameter for digitoxin dosing, which was not expected because of the pharmacokinetic properties of digitoxin and not evident from retrospective data. This underlines the importance of prospective, randomized, and blinded clinical studies and is of particular relevance in clinical practice because a significant number of heart failure patients have concomitant impairment of renal function.

Based on recent analyses, cardiac glycosides still may be an important option for the treatment of patients suffering from HFrEF [22–24]. Cardiac glycosides may be, in particular, valuable in advanced heart failure with highly symptomatic patients despite exploited modern pharmacotherapy and device therapy or if applicability of these therapies is limited due to relevant comorbidities, e.g., hypotension and impaired renal function. DIGIT-HF will provide important evidence, whether the cardiac glycoside digitoxin improves prognosis and reduces hospital admissions for worsening heart failure in advanced chronic HFrEF, which will significantly be supported by the digitoxin dosing recommendations based on the DIGIT-HF trial data presented in this manuscript. Because DIGIT-HF included a substantial number of patients with atrial fibrillation, the digitoxin dosing score might also work in atrial fibrillation to achieve safe and effective rate control irrespective of left ventricular ejection fraction.

## Conclusion

A digitoxin dosing score comprising information on age, sex, BMI, and eGFR was derived and validated in patients with HFrEF randomized to digitoxin treatment in the DIGIT-HF trial. The new digitoxin dosing score advises, whether a patient should be started with a low dose of 0.05 mg digitoxin o.d. The digitoxin dosing score can easily be obtained in clinical routine and enables simple and safe initial digitoxin dosing in patients with HFrEF.

## Data Availability

Data is available to the scientific community on request. All data requests should be submitted to the corresponding author for consideration. Access to anonymised data might be granted following review. However, because we have to ensure blinding and integrity of the DIGIT-HF trial, we cannot provide access to original trial data beyond as currently presented in this manuscript before the end of the trial.
